# Prevalence of asymptomatic *Plasmodium* infections with sub-microscopic parasite densities in the northwestern border of Thailand: a potential threat to malaria elimination

**DOI:** 10.1186/s12936-018-2476-1

**Published:** 2018-09-12

**Authors:** Jetsumon Sattabongkot, Chayanut Suansomjit, Wang Nguitragool, Jeeraphat Sirichaisinthop, Saradee Warit, Montip Tiensuwan, Sureemas Buates

**Affiliations:** 1 Mahidol Vivax Research Unit, Faculty of Tropical Medicine, Mahidol University, Bangkok, Thailand; 2 Department of Microbiology, Faculty of Science, Mahidol University, Bangkok, Thailand; 3 Department of Molecular Tropical Medicine and Genetics, Faculty of Tropical Medicine, Mahidol University, Bangkok, Thailand; 4 Bureau of Vector Borne Diseases, Pra Phuttabhat, Saraburi Thailand; 5 Tuberculosis Research Laboratory, Medical Molecular Biology Research Unit, BIOTEC, National Science and Technology Development Agency, Thailand Science Park, Pathum Thani, Thailand; 6 Department of Mathematics, Faculty of Science, Mahidol University, Bangkok, Thailand

**Keywords:** Prevalence, Asymptomatic, Sub-microscopic, Malaria, Thailand

## Abstract

**Background:**

Asymptomatic infections with sub-microscopic *Plasmodium* serve as a silent reservoir of disease, critical to sustaining a low level of remanent malaria in the population. These infections must be effectively identified and targeted for elimination. The sensitivity of light microscopy, the traditional method used for diagnosing *Plasmodium* infections, is frequently insufficient for detecting asymptomatic infections due to the low density of parasitaemia. The objective of this study was to explore the current prevalence of asymptomatic sub-microscopic *Plasmodium* carriages to evaluate the parasite reservoir amongst residents from 7 hamlets in Tak Province in northwestern Thailand using a highly sensitive molecular method.

**Methods:**

Malaria infection was screened in a real-world setting from 3650 finger-prick blood specimens collected in a mass cross-sectional survey using light microscopy and loop-mediated isothermal amplification (LAMP). LAMP results were later confirmed in a laboratory setting in Bangkok using nested PCR, restriction enzyme digestion and DNA sequencing. The association of malaria infection with demographic factors was explored.

**Results:**

Parasite prevalence was 0.27% (10/3650) as determined by microscopy. Sub-microscopic infection prevalence was 2.33% (85/3650) by LAMP. Of these, 30.6% (26/85) were infected with *Plasmodium falciparum*, 52.9% (45/85) with *Plasmodium vivax*, 2.4% (2/85) with *Plasmodium malariae*, 4.7% (4/85) with mixed *P. falciparum* and *P. vivax*, and 9.4% (8/85) had parasite densities too low for species identification. Asymptomatic carriages (T < 37.5 **°**C) accounted for 95% (76/80) of all sub-microscopic cases with the highest prevalence occurring in the subjects 31–45 years of age (*p *≤ 0.035). Participants working on plantations or as merchants had an increased infection risk. Evaluation by microscopy identified 10.53% (10/95) of all *Plasmodium* infected participants.

**Conclusion:**

Participants carrying asymptomatic *Plasmodium* infections with sub-microscopic parasite densities are considerable in this area. These findings provide the true disease burden and risk factors in this region. This information helps to direct policy makers towards better schemes and delivery of targeted interventions. Moreover, this is the first study to use LAMP in mass screening for sub-clinical and sub-microscopic infections in a field setting in Thailand. LAMP proves to be a sensitive and field-deployable assay suitable for national malaria control screening campaigns.

## Background

Based on the world malaria report in 2017 from 91 countries and areas with ongoing malaria transmission, the progress of malaria control efforts has stalled after an unprecedented session of successfulness in worldwide malaria control [1]. In 2016, there were approximately 216 million malaria cases, an increase of 5 million cases compared to 2015. In 2016, the number of deaths was 445,000 worldwide which is a similar number to the previous year. Of these deaths, 92% occurred in the African Zone, followed by 6% in the Southeast Asian Zone, as well as 2% in the Eastern Mediterranean Zone [1].

According to malaria statistics for Thailand in 2017, malaria control efforts in Thailand have been highly successful, resulting in an 82.2% decrease in clinical cases over the last 10 years since 2007 from 63,354 to 11,263 cases [2]. Nonetheless, malaria is still endemic in some parts of Thailand. The distribution of malaria in Thailand is patchy and can be typified as ‘border malaria’ and ‘forest malaria’, with highest transmission along international borders and in the hills, forests or forest fringes [2]. The borders where malaria outbreaks occurred were along the western border with Myanmar (29.1%), the southern border with Malaysia (31.3%), the eastern border with Cambodia (7%), and to a lesser degree, the eastern border with Laos (2.1%) [3]. Resultantly, malaria transmission along these four international borders accounted for 69.5% of the cases of malaria in Thailand [3]. Historically, the western border with Myanmar has had the highest prevalence of malaria in Thailand [2]. This area has been the focus of vigorous malaria control programmes for decades. Consequently, parasite prevalence in this area has declined and the highest parasite prevalence in the country is now in the southern border with Malaysia [3]. Currently, there are 6 *Plasmodium* species, *Plasmodium falciparum*, *Plasmodium vivax*, *Plasmodium malariae*, *Plasmodium ovale curtisi*, *Plasmodium o. wallikeri* as well as *Plasmodium knowlesi* that can infect humans. Of these, two species, *P. vivax* (76.70%) and *P. falciparum* (15.23%) are the predominant species found in Thailand [3].

As the malaria burden still poses serious challenges, the Thai Government has declared a national malaria elimination goal of 2024 [4]. To accomplish this goal, the priorities under the latest strategic plan include increased detection of both symptomatic and asymptomatic malaria together with accurate treatment. Detection of all infected persons, including asymptomatic carriers with low and sub-microscopic parasite densities is critical to the elimination of malaria, as these carriers harbour a parasite reservoir in the population which can still potently be transmitted to mosquitoes and contribute to human transmission [5]. In Thailand, light microscopy analysis of blood smears is the gold standard in malaria diagnosis. However, microscopy is known for being insensitive at low-level parasitaemia. Therefore, the actual prevalence of the malaria parasite should be underestimated. As assessed via standardization with a thick blood film spiked with the parasite, the limit of detection of microscopy for detecting parasitaemia is between 10 and 100 parasites/μL (10,000–100,000 parasites/mL) [6, 7]. Therefore, there is a great need for more accurate methods of detection for community-based studies quantifying asymptomatic and very low-density *Plasmodium* infections for improving malaria elimination strategies. In addition to being a highly sensitive diagnostic, the ideal method needs to be readily field-applicable for pre-elimination field screening teams.

The study site in this current study is located in Tak, a Myanmar-border province, previously the location of Thailand’s highest malaria burden. In the past few years, however, this province experienced a substantial decrease in transmission [8, 9]. However, based on microscopy evaluation, a yearly parasite incidence of 5.04 cases/1000 was found in 2017. Tak still represents a malaria hotspot in Thailand [3]. For a mass screening survey in this region, most of the research used a quantitative polymerase chain reaction (qPCR) method to detect asymptomatic submicroscopic *Plasmodium* infections. This method had to be conducted in Bangkok [10, 11]. Up until now, no molecular-based studies have been done in a field setting for mass screening in this area of Thailand.

A newer molecular method, loop-mediated isothermal amplification (LAMP) has been shown to have an estimated detection limit of 1 parasitized red blood cell (RBC)/500 μL of blood [12]. LAMP is a ground-breaking gene amplification technique, and has recently been used in field settings to evaluate malaria prevalence [13–17]. Reverse transcription (RT)-LAMP has been shown to be roughly 100-fold more sensitive than reverse transcription (RT)-qPCR [18]. LAMP is more accessible in field settings than PCR since it can be deployed outside reference laboratories. LAMP can be performed without a thermal cycler and has diagnostic accuracy comparable to PCR, without demanding complex equipment for sample processing or result analysis. Therefore, LAMP offers a simple, reliable, sensitive, and cost-effective tool for significantly improving the detection of asymptomatic *Plasmodium* infections with low-density parasites, that otherwise go undetected by microscopy.

To date, there have been no studies done to determine the prevalence of asymptomatic *Plasmodium* infections with sub-microscopic and low-density malaria reservoirs by LAMP in a real-world field setting of communities in Thailand. Accordingly, a cross-sectional survey was conducted in 3650 people living in malaria-endemic communities in Tak Province, Thailand. Here, a malariometric survey performed in the field by LAMP assay to determine the current epidemiology of asymptomatic sub-microscopic carriers was described. This study explored the risk factors associated with residual malaria parasitaemia amongst local residents, for a more comprehensive understanding of the malaria epidemiology.

## Methods

### Study site description

**Fig. 1:**
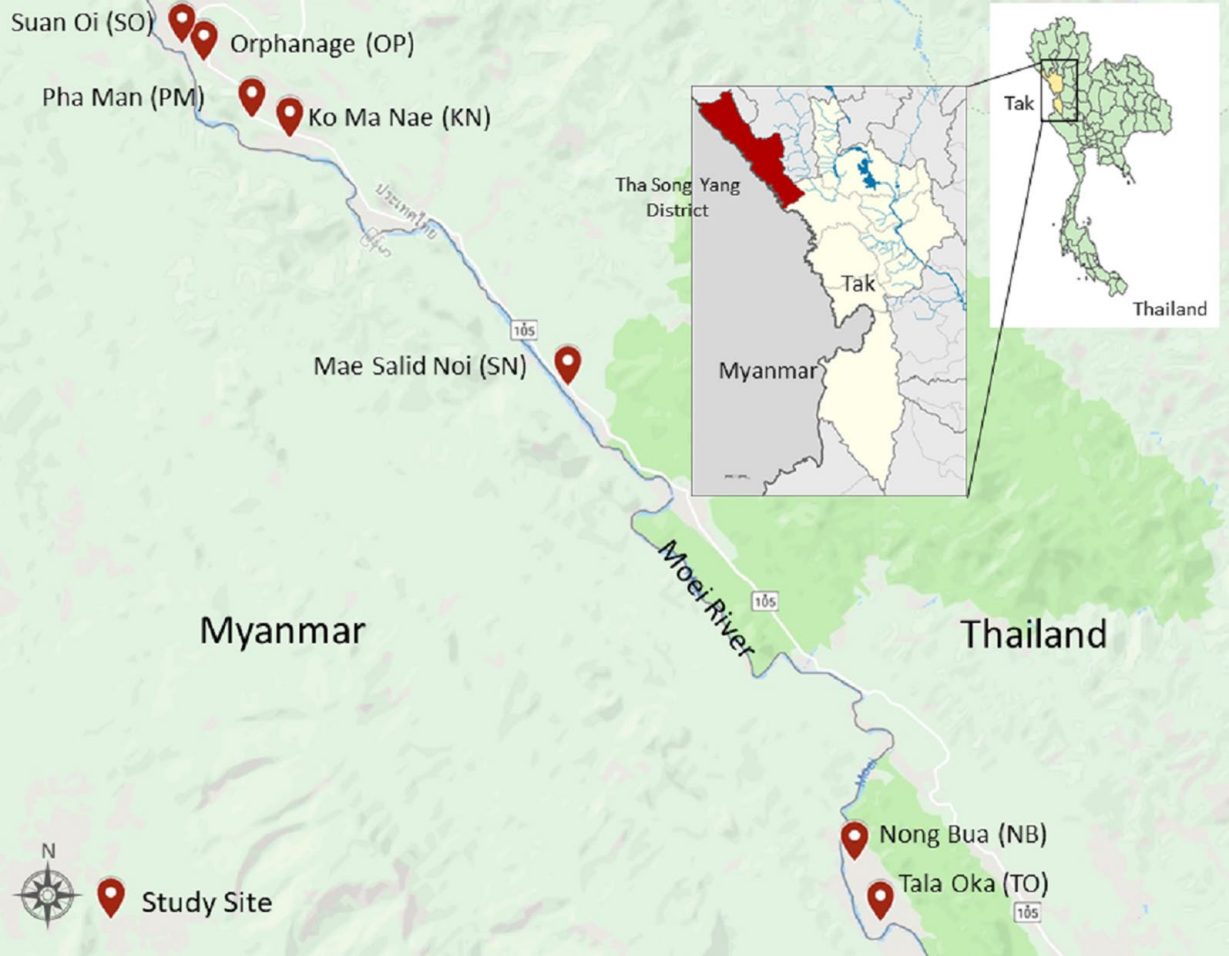
Study sites in Tha Song Yang district, Tak Province, Thailand

The community-based cross-sectional survey was conducted in Tha Song Yang district, Tak Province, northwestern Thailand (Fig. 1). Tha Song Yang is approximately 595 km from the capital. It is located at 17°13′36″N, 98°13′30″E with 1010 m above the sea level. It is situated in the northwest region of Tak, on the Moei River bank near the Myanmar border. The climate is tropical with an average temperature of 26.4 °C. The rainy season is between May and October with an average yearly rainfall of 1540 mm. The inhabitants of this area are approximately 30% Thai nationality and 70% ethnic minorities. Normally, there are two peaks of malaria transmission in western Thailand, including Tak Province. One peak is at the beginning of the rainy season (May–August) and the other is at the end of the rainy season (October) [19]. *Plasmodium vivax* and *P. falciparum* are predominant in this region, although all human malaria parasites, as well as the simian malaria species *P. knowlesi*, can be found [20]. There are 6 sub-districts in Tha Song Yan, which are further sub-divided into 56 villages. Among these, 7 hamlets (Nong Bua, Tala Oka, Mae Salid Noi, Suan Oi, Pha Man, Ko Ma Nae, and Orphanage) were chosen for the study to conduct a community-based cross-sectional survey of *Plasmodium* prevalence.

### Data and sample collection

**Fig. 2:**
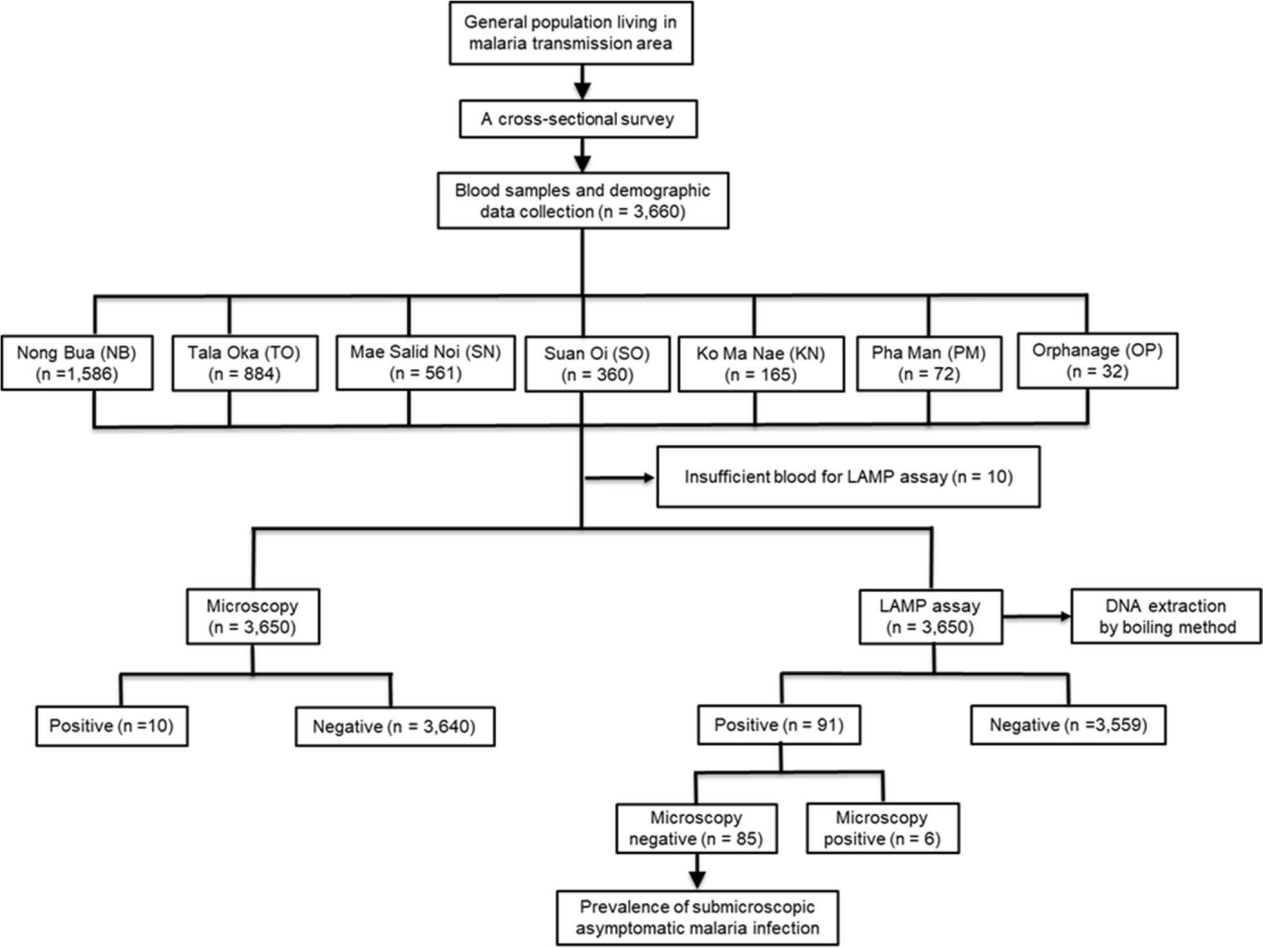
Study design for determination of *Plasmodium* carriages in Tha Song Yang district, Tak Province, Thailand

A mass cross-sectional study was conducted to determine the prevalence of asymptomatic sub-microscopic malaria carriages from 13 to 27 July, 2015. An ethical consent for this study was provided by an ethical committee, Department of Disease Control, Ministry of Public Health, Nonthaburi, Thailand (Ref No 7/54-479). All individuals and all age groups were invited to participate in the study. Signed informed consents were obtained from all participants before enrolment. Interviews were performed with parents and/or legal guardians for participants 7 years or younger. A selection criteria for participation was the absence of anti-malarial drug treatment within the previous 2 weeks. The participant’s history of previous malaria infection, body temperature, occupation, age and gender, were recorded at the time of enrolment. The study design for determination of *Plasmodium* carriages is shown in Fig. 2.

EDTA microtainer tubes were used to collect finger-prick blood samples from 3660 participants who met the inclusion criteria. However, 10 blood samples were excluded from the study since the blood was not sufficient to perform LAMP analysis. For each of the finger-prick blood sample, a portion of the blood was utilized directly to prepare thick and thin blood smears for microscopic analysis. For screening by a *Plasmodium* genus-specific LAMP assay in the field, 50 μL of blood was used. LAMP genus-positive blood samples were further characterized by a LAMP species-specific assay in the field. The remainder of blood samples was kept at − 80 °C for repeating LAMP analyses in a laboratory setting in Bangkok. Results of the repeated LAMP reactions were later confirmed using a combination of nested PCR and restriction enzyme digestion, followed by DNA sequencing.

### Conventional microscopy

Thick and Thin blood smear preparation was done on one slide, air-dried, fixed with methanol (thin blood smears) and stained for 10 min with 10% Giemsa. Thick blood films were examined immediately under a light microscope (1000× magnification) by clinical staff in the field as per routine malaria diagnosis. *Plasmodium* density obtained from thick smear analysis was interpreted as the parasite number detected per 500 white blood cells (WBCs) and was converted into the parasite number/ml of blood presuming a WBC count of 8000 WBCs/μL of blood [21]. If no parasites were detected after 500 WBCs were counted, the thick blood smear was classified as negative. The blood smears were re-read in the field by an expert microscopist, of skill level 1 or 2 following the WHO competency assessment protocol, to confirm the result. All microscopists were blinded from the LAMP results. Thin blood smears were used for determination of the parasite species via counting the number of parasites detected per 5000 RBCs.

### Genomic DNA (gDNA) extraction

For the LAMP assay, a simple boiling technique was used in this study to extract genomic DNA [16, 17]. Briefly, 50 µL of EDTA treated whole blood was added into a 1.5 ml-microcentrifuge tube containing 150 µL of nuclease-free distilled water, boiled for 5 min at 95 °C and centrifuged for 3 min at 2046×*g*. For each LAMP reaction, 100 µL of the clear supernatant was transferred to a new clean tube and 5.5 µL of the supernatant was used immediately. Based on the observation, the supernatant containing gDNA could be kept at − 80 °C for not more than 7 days without compromising stability.

For nested PCR, DNA extraction was done from frozen EDTA blood samples using a NucleoSpin^®^ Blood mini kit (Macherey–Nagel GmbH, Germany) according to the manufacturer’s protocol. For nested PCR analysis, 5 µL of the eluted DNA was used directly.

### Detection of *Plasmodium* genus- and species-specific genes by LAMP

LAMP primer sets targeting 18S ribosomal RNA (*18srRNA*) as described previously by Han et al. [22] were used for detecting all *Plasmodium* spp. and 4 human *Plasmodium* parasites including *P. falciparum*, *P. vivax*, *P. malariae*, and *P. ovale* [22]. The primer set used for quantification of the apical membrane antigen-1 (*AMA*-*1*) gene, which is found during the late schizont stage of all *Plasmodium* parasites, was used to identify *P. knowlesi* as described by Lau et al. [23]. A Loopamp DNA amplification kit (Eiken Chemical Co., Ltd., Tokyo, Japan) was used to perform the LAMP reaction. The 25-µL LAMP reaction mixture contained 1.6 µM of each forward inner primer (FIP) and backward inner primer (BIP) for *Plasmodium* genus, *P. falciparum*, *P. ovale*, and *P. knowlesi* and 2.4 µM of each FIP and BIP for *P. vivax* and *P. malariae*, 0.2 µM of each forward outer primer (F3) and backward outer primer (B3), 0.8 µM of each loop primer forward (LPF) and loop primer backward (LPB) 12.5 µL of 2× reaction mix, 1 µL of *Bst* DNA polymerase and 5.5 µL of DNA template. The LAMP reaction mixture was incubated in a Loopamp real-time turbidimeter (LA-320C, Eiken Chemical Co., Ltd., Tokyo, Japan) or in a water bath for 90 min at 60 °C and for 5 min at 80 °C for enzyme inactivation. A negative control was included in each run. The LAMP reaction was assessed by the naked eye for endpoint detection or by a Loopamp real-time turbidimeter (LA-320C) for real-time detection. LAMP reactions for *Plasmodium* genus and species were interpreted as positive when an obvious increase in turbidity was detected by the naked eye or by real-time turbidimeter readings. The results were interpreted as valid when the turbidity was absent in the negative control.

### Detection of *Plasmodium* genus- and species-specific genes by nested PCR

The species-specific nucleotide sequences of the 18S ribosomal RNA *(18SrRNA)* genes of *P. falciparum, P. vivax, P. malariae,* and *P. ovale* were amplified in two successive rounds of PCR as described previously [24]. For the first round of PCR, primers used were specific for all *Plasmodium* spp. In the second round of PCR, the primers were specific for 4 human malaria species (*P. falciparum*, *P. vivax*, *P. malariae*, and *P. ovale*). The PCR reaction was carried out in a 25-µL reaction volume consisting of 12.5 µL GoTaq^®^ Green Master Mix (Promega, USA), 0.4 µM of each forward and reverse primers for *Plasmodium* genus, and 5 µL of template DNA. In the second round of PCR, the PCR product from the first round was diluted 10-fold in nuclease-free distilled water, and 5 µL was added to a new PCR reaction mixture containing the same concentration of species-specific primers as the genus-specific primers in the first round. For *P. knowlesi*, 2 sets of primers were used to amplify the small sub-unit ribosomal RNA (*ssrRNA*) gene. *Plasmodium*-specific primers (*rPLU1* and *rPLU5*) were utilized in the first-round [25]. *Plasmodium knowlesi*-specific primers (*Pmk8* and *Pmkr9*) were utilized in the second round [26]. Amplification was done in a 25 µL reaction mixture containing 5 µL of template DNA, 0.25 µM of each primer (*rPLU1* and *rPLU5*), 12.5 µL of GoTaq^®^ Green Master Mix (Promega, USA). In the first PCR, the *ssrRNA* gene was amplified following the conditions as described previously [25]. In the second PCR, 5 µL of the first PCR product was utilized as the template DNA. The conditions and concentrations of the second PCR were similar to the first one, except the annealing temperature was set at 58 °C [23]. The amplicons were analysed by electrophoresis in a 2% agarose gel stained with ethidium bromide and visualized under a UV light.

### Confirmation of LAMP products by restriction enzyme digestion

For further confirmation, LAMP amplified products were validated by restriction enzyme digestion, followed by comparison with the restriction map of the target sequences of each of the LAMP products. For restriction enzyme analysis, *DdeI* was used for analysis of *Plasmodium* genus-specific LAMP products. *HpyCH4V* was used for *P. falciparum*, *P. vivax*, and *P. malariae*. *AluI* was used for *P. ovale*. LAMP products were digested with restriction enzymes at 37 °C for overnight and were incubated at 65 °C for 20 min to inactivate the enzyme. Digested LAMP products were analyzed by electrophoresis in a 3% agarose gel, stained with ethidium bromide and visualized under a UV light.

### DNA sequencing

In order to confirm the *Plasmodium* genus and species of the LAMP-positive blood samples, the LAMP products were used as template DNA for PCR amplification using LF and LB primers. The products were run on an agarose gel, stained with SYBR safe DNA gel stain (Invitrogen, USA), and the specific band was cut and purified with a QIAquick Gel Extraction kit (Qiagen, Germany). The purified amplicons were cloned into the pGEM^®^-T Easy Vector (Promega, USA) and sequenced using an ABI3730XL DNA Analyzer (Macrogen, Korea) with 2 universal primers (T7 and SP6). The sequences were aligned with standard sequences of the *Plasmodium 18srRNA* gene.

### Statistical analysis

Data analysis of factors associated with *Plasmodium* infections was done using binary logistic regression. Chi square (χ^2^) test with 95% CI was used for *p*-value calculation. The SPSS 17.0 statistical software package (SPSS, Inc, Chicago, USA) was used for statistical analysis.

## Results

### Study population demographic characteristics

The mass cross-sectional survey was conducted between 13 and 27 July, 2015. The blood samples were collected with informed consents from 3660 residents living in 7 hamlets in Tha Song Yang district, Tak Province, Thailand. Ten samples had to be excluded from the study since there was insufficient blood to perform a LAMP reaction. Of these 3650 participants, 2035 (55.75%) were females and 1615 (44.25%) were males (Table 1). The age of the study population ranged from 1 month to 99 years. Overall, the median age was 15 years (IQR = 28 years) and the mean age was 22.63 years (± SD = 19.34). The predominant participants were the age 6–15 years accounted for 34.22% (1249/3650) of all participants. The participants which the age > 60 years were the minority group accounted for 5.7% (208/3650) of the study population. More than half (58.68%, 2142/3650) of the participants were foreigners and 41.32% (1508/3650) had Thai citizenship. Participant’s body temperature was recorded during blood sample collection. Asymptomatic malaria in this study was classified as a malaria infection found in afebrile participants with axillary temperature < 37.5 °C at the time of the survey with no presence of fever within the previous 2 days. Participants with body temperature of < 37.5 °C accounted for 94.83% (3411/3597) of the study population, and only 5.17% (186/3597) had a fever (≥ 37.5 °C). Three febrile participants were blood smear negative but were later found to be LAMP positive. Using LAMP, one febrile participant (a foreigner) with a body temperature of 38 °C had *P. vivax* infection. The other 2 participants with body temperature of 38.1 °C (Thai) and 39 °C (foreigner) had *P. vivax* infection and *P. falciparum* infection, respectively. The most frequent occupation among participants (39.45%, 1440/3650) was student. Among the 7 study sites, Nong Bua had the highest population number (43.26%, 1579/3650) followed by Tala Oka, Mae Salid Noi, Suan Oi, Ko Ma Nae, Pha Man, and Orphanage, respectively.

**Table 1:** Characteristics of the study population

Characteristics	Number	%
Study sites (n = 3650)		
Nong Bua	1579	43.26
Tala Oka	883	24.19
Mae Salid Noi	560	15.34
Suan Oi	359	9.84
Ko Ma Nae	165	4.52
Pha Man	72	1.97
Orphanage	32	0.88
Gender (n = 3650)		
Male	1615	44.25
Female	2035	55.75
Age		
Mean = 22.63 years		
Range = 1 month–99 years		
Median = 15 years		
± SD = 19.34		
IQR = 28		
Age (n = 3650), years		
≤5	636	17.42
6–15	1249	34.22
16–30	640	17.53
31–45	579	15.86
46–60	338	9.26
> 60	208	5.70
Body temperature (n = 3597), °C		
≥ 37.5	186	5.17
< 37.5	3411	94.83
Not determine^a^	53	
Citizenship (n = 3650)		
Thai citizenship	1508	41.32
Foreigner	2142	58.68
Occupation (n = 3650)		
Student	1440	39.45
Child	633	17.34
Hireling/labour	781	21.40
Plantation	493	13.51
Housekeeper/housewife	74	2.03
Merchant	66	1.81
Other (e.g., cripple, retired, monk, government officer)	163	4.47

^a^53 samples were excluded since body temperature data was not available

### LAMP and microscopy-based determination of *Plasmodium* carriages

Using LAMP, a total of 3650 blood samples were screened in the field. For LAMP genus-specific screening of sub-microscopic malaria infections, 50 μL of each finger prick blood sample was used directly for DNA extraction by a boiling method and 5.5 µL of freshly prepared supernatant was used immediately. Approximately 200 µL of the remaining blood was kept at 4 °C while waiting for the LAMP genus-specific results. For LAMP genus-positive samples, the supernatants were kept at − 80 °C for overnight before being used for LAMP species-specific analysis. For samples deemed positive in the genus-specific LAMP assay or both genus- and species-specific LAMP assays, 200 µL of the remaining blood samples was kept at − 80 °C for LAMP confirmation in the laboratory.

Of the 3650 blood samples screened in the field, LAMP successfully amplified *Plasmodium* DNA in 2.58% (94/3650). Among these LAMP-positive samples, 0.16% (6/3650) were also positive by microscopy. Thus, LAMP identified a substantially higher number of *Plasmodium* carriages (2.49%; 88/3650) as shown in Table 2. LAMP results in the field were later repeated in a laboratory setting in Bangkok, and were confirmed using a combination of nested PCR, restriction enzyme digestion, and DNA sequencing. After confirmation of 88 LAMP-positive samples, there were 3 discordant results composed of 1 sample positive for *P. falciparum* and 2 samples positive for *P.* vivax by LAMP. These samples were negative by restriction enzyme digestion. In summary, overall the prevalence of sub-microscopic infections as determined by LAMP was 2.33% (85/3650). Details of the prevalence of sub-microscopic *Plasmodium* infections detected by LAMP in the 7 hamlets are presented in Table 3.

**Table 2:** Detailed comparison of LAMP and nested PCR combined with restriction enzyme digestion for *Plasmodium* identifications

Parasite(s) detected by each method^a^ (no. of samples; n = 3650 blood samples)	
LAMP	Nested PCR combined with RE digestion of LAMP products^b^
*P. falciparum* (27)	*P. falciparum* (26), *negative (1)*^c^
*P. vivax* (47)	*P. vivax* (45)*, negative* (2)^d^
*P. falciparum *+* P. vivax* (4)	*P. falciparum *+* P. vivax* (4)
*P. malariae* (2)	*P. malariae* (2)
*Plasmodium* spp.^e^ (8)	*Plasmodium* spp. (8)
Negative (3562)	Negative (3562)

^a^Each row displays the results obtained from identical blood samples. Discordant results between LAMP and nested PCR combined with restriction enzyme digestion are showed in italicface

^b^Genus and species-specific LAMP products were digested with restriction enzyme to confirm the specificity of LAMP products

^c^Positive for *P. falciparum* by LAMP but negative after restriction enzyme digestion of LAMP products

^d^Positive for *P. vivax* by LAMP but negative after restriction enzyme digestion of LAMP products

^e^Positive for genus-specific LAMP but negative for species-specific LAMP

**Table 3:** Prevalence of *Plasmodium* carriages based on microscopy and LAMP

Hamlet	Nong Bua	Tala Oka	Mae Salid Noi	Suan Oi	Ko Ma Nae	Pha Man	Orpha-nage	Total
Total population (n)	1579	883	560	359	165	72	32	3650
Microscopy, n (%)	2^a^ (0.13%)	0 (0%)	1 (0.18%)	3^b^ (0.84%)	1 (0.61%)	1 (1.39%)	2 (6.25%)	10 (0.27%)
LAMP, n (%)	49 (3.1%)	12 (1.36%)	5 (0.89%)	21 (5.85%)	1 (0.61%)	1 (1.39%)	2 (6.25%)	91 (2.49%)
Sub-microscopy, n (%)	49 (3.1%)	12 (1.36%)	4 (0.71%)	20 (5.57%)	0 (0%)	0 (0%)	0 (0%)	85 (2.33%)

^a^Both samples were positive by microscopy but negative by LAMP

^b^Only one sample was positive by microscopy and LAMP

Thick and thin blood films from the 3650 blood samples were analysed by Giemsa-stained smear examination by local staff in the field and later confirmed by an expert microscopist. After the initial and second round of microscopy, only 10 samples were found positive for *Plasmodium* infections (3 in Suan Oi, 2 in Nong Bua and Orphanage, and 1 in Mae Salid Noi, Ko Ma Nae, and Pha Man), representing an overall prevalence of 0.27% (10/3650) (Table 3**)**. These 10 samples classified as positive by microscopy originated from 8 male and 2 female subjects. Among 10 samples positive by microscopy, 4 samples were negative by LAMP.

As shown in Table 4, the majority of sub-microscopic *Plasmodium* carriages was a single *P. vivax* infection, accounting for 52.9% (45/85) of the cases. A single *P. falciparum* infection was present in 30.6% (26/85) of the subjects. A single *P. malariae* infection accounted for 2.35% (2/85) of the subjects. Mixed infections between *P. falciparum* and *P. vivax* accounted for 4.75% (4/85) of the cases and 9.41% (8/85) of the cases identified as *Plasmodium* genus-positive.

**Table 4:** Details of sub-microscopic *Plasmodium* species

Hamlet	Nong Bua	Tala Oka	Mae Salid Noi	Suan Oi	Ko Ma Nae	Pha Man	Orpha-nage	Total
Total population (n)	1579	883	560	359	165	72	32	3650
Sub-microscopy n (%)	49 (3.1%)	12 (1.36%)	4 (0.71%)	20 (5.57%)	0 (0%)	0 (0%)	0 (0%)	85 (2.33%)
*Plasmodium* spp.								
* Pv*	22 (44.9%)	8 (66.67%)	1 (25%)	14 (70%)	0 (0%)	0 (0%)	0 (0%)	45 (52.9%)
* Pf*	17 (34.7%)	3 (25%)	3 (75%)	3 (15%)	0 (0%)	0 (0%)	0 (0%)	26 (30.6%)
* Pm*	2 (4.08%)	0 (0%)	0 (0%)	0 (0%)	0 (0%)	0 (0%)	0 (0%)	2 (2.35%)
* Pf *+ *Pv*	4 (8.16%)	0 (0%)	0 (0%)	0 (0%)	0 (0%)	0 (0%)	0 (0%)	4 (4.75%)
* P.* spp.	4 (8.16%)	1 (8.33%)	0 (0%)	3 (15%)	0 (0%)	0 (0%)	0 (0%)	8 (9.41%)

*Plasmodium.* spp.: *Plasmodium* genus positive but species negative

*Pv*, *P. vivax; Pf, P. falciparum; Pm, P. malariae*

### Demographic characteristics of the study population associated with sub-microscopic *Plasmodium* infections

Detailed analysis of the association of the demographic characteristics of the study population and sub-microscopic *Plasmodium* infections is shown in Table 5. Although the prevalence of sub-microscopic malaria infections was higher in female (2.6%) than male (1.98%) subjects, no significant difference was observed between these two groups. Most LAMP-positive sub-microscopic *Plasmodium* infections were found in adults and were significantly higher in subjects aged 31–45 years (3.28%, 19/579) *p *≤ 0.035. Among the positive samples, the youngest and oldest subjects were 1 and 78 years of age, respectively. Most of the sub-microscopic malaria infections (95%, 76/80) were asymptomatic, since indications of malaria-like symptoms, such as headache, chills and fever were not observed. The majority of the sub-microscopic cases of malaria occurred in the participants with no Thai citizenship (60%, 51/85). Furthermore, a strong association between asymptomatic *Plasmodium* infections and patient occupations was observed in this study. The risk of acquiring asymptomatic sub-microscopic *Plasmodium* infections significantly increased in individuals working on the plantation (*p *≤ 0.013) or as merchants (*p *≤ 0.015). The odds ratios of plantation and merchant occupations were approximately 2 and 4 times greater than other occupations, respectively. Among 7 hamlets, the prevalence of asymptomatic *Plasmodium* infections was significantly high in Nong Bua (3.1%; 49/1579) *p *≤ 0.004 and Suan Oi (5.57%; 20/359) *p *≤ 0.05.

**Table 5:** Demographic characteristics of the study population associated with asymptomatic sub-microscopic *Plasmodium* infections

Characteristics	Total samples	Prevalence of sub-microscopic infections^a^, n (%)	Odds Ratio (95% CI)	P value^b^
Gender				
Male	1615	32 (1.98%)	1.32 (0.85–2.05)	0.223
Female	2035	53 (2.6%)		
Age (years)				
≤ 5	636	9 (1.42%)	Ref. group	–
6–15	1249	25 (2%)	1.43 (0.66–3.07)	0.366
16–30	640	18 (2.81%)	2.02 (0.9–4.54)	0.087
31–45	579	19 (3.28%)	2.36 (1.06–5.27)	0.035*
46–60	338	9 (2.66%)	1.91 (0.75–4.85)	0.175
> 60	208	5 (2.4%)	1.71 (0.57–5.17)	0.339
Body temperature^c^ (°C)				
≥ 37.5	186	4 (2.15%)	0.96 (0.35–2.66)	0.94
< 37.5	3411	76 (2.23%)		
Citizenship				
Thai citizenship	1508	34 (2.25%)	1.06 (0.68–1.64)	0.795
Foreigner	2142	51 (2.38%)		
Study site				
Nong Bua	1579	49 (3.1%)	4.45 (1.59–12.38)	0.004*
Tala Oka	883	12 (1.36%)	1.91 (0.61–5.96)	0.264
Mae Salid Noi	560	4 (0.71%)	Ref. group	–
Suan Oi	359	20 (5.57%)	8.26 (2.79–24.37)	0*
Ko Ma Nae	165	0 (0%)	0	1
Pha Man	72	0 (0%)	0	1
Orphanage	32	0 (0%)	0	1
Occupation				
Student	1440	32 (2.22%)	1.58 (0.75–3.33)	0.23
Hireling/labour	781	18 (2.32%)	1.64 (0.73–3.68)	0.229
Child	633	9 (1.42%)	Ref. group	–
Plantation	493	19 (3.85%)	2.78 (1.24–6.19)	0.013*
Housekeeper	74	0 (0%)	0	1
Merchant	66	4 (6.06%)	4.47 (1.34–14.92)	0.015*
Other (e.g., cripple, retired, monk, government officer)	163	3 (1.84%)	1.29 (0.35–4.85)	0.698

*Statistically significant difference

^a^Sub-microscopic infection was defined when the sample was LAMP-positive but microscopy negative

^b^P values were obtained by binary logistic regression and is significant at < 0.05

^c^53 samples were excluded since the body temperature data was not available

## Discussion

In 2016, Thailand had approximately 11,522 malaria cases, or 0.17 cases/people [27], and was considered a region of low malaria transmission. Currently, the country is in ‘control’ phase and proposes to achieve malaria elimination by the year 2024 based on World Health Organization efforts, and the National Malaria Strategy [28]. This is a part of a wider goal for a malaria-free Asia–Pacific by the year 2030, proclaimed by the Asia–Pacific Leaders Malaria Alliance at the 9th East Asia Summit in 2014. To reach such a goal, rapid identification (including parasite speciation) of asymptomatic and symptomatic individuals as well as an accurate and proper treatment of symptomatic infections and malaria reservoirs are critical to accomplish. Individuals with asymptomatic sub-microscopic malaria infections are silent reservoirs of the parasite and pose a serious challenge to disease control efforts due to their ability to maintain transmission within the population at a low level.

The studied area in this current study is one of the most malaria-endemic regions of Thailand. Although there has been a marked reduction in malaria transmission, Tak Province in 2017 was still classified as a high transmission area based on the WHO 2016 index, with an estimated annual incidence of 5.04 cases/1000 people [27]. In 2017, an estimated annual incidence in Thailand was 0.17 cases/1000. The estimated annual incidence in Tak Province was approximately 29 times the national average [3]. This estimate in incidence was obtained by the standard malaria diagnosis method of light microscopy analysis of blood smears which lacks sensitivity enough for detecting low-level parasitaemia [6]. To get the actual number of malaria incidence in Thailand, molecular techniques which are more sensitive have been used as an alternative method.

Quantitative polymerase chain reaction (qPCR) has been used to detect a number of asymptomatic sub-microscopic malaria infections in this study area [10, 11]. Although this assay is sensitive, it must be performed in a laboratory setting. qPCR is not applicable for use in the field. To date, no molecular methods were conducted in real-life field settings for the malaria mass screening in this area of Thailand. The current study is the first mass cross-sectional survey of sub-clinical and sub-microscopic *Plasmodium* infections in Thailand conducted in field clinics using a LAMP method. LAMP results revealed that there were 3 samples positive by LAMP but negative after LAMP product confirmation by restriction enzymes digestion. These false positives could be due to haemolysis or high levels of lipid in the plasma of the blood samples. Moreover, 4 samples were positive by microscopy but negative by LAMP. In these samples, the parasite density ranged between 16 and 1440 parasites/µL, which could be detected by LAMP. Thus, there should be other causes for obtaining LAMP-negative results including a lower efficiency of template DNA preparation or DNA degradation during the preparation process. Overall, the LAMP method performed in the real-world field setting demonstrated reliable results. In this study, LAMP proves to be easy to operate and simple to adapt for use in any environment and field condition, and can be used as a reliable method for discerning the hidden malaria reservoir in various communities.

This current study found that malaria infection rates estimated by LAMP in the mass blood survey of this community were 8.6 times higher than those estimated by an expert microscopist. At the time of this survey, the prevalence of *Plasmodium* infections was 2.6% (95/3650) and over 85.6% were asymptomatic sub-microscopic infections. This suggests that there is a substantial proportion of *Plasmodium*-infected individuals with both asymptomatic and sub-microscopic densities in this area. This current finding is in agreement with the results from the previous study by Baum et al. conducted in the same area using qPCR, which found that the prevalence of asymptomatic and sub-microscopic *Plasmodium* infections in the population was 3.0 and 90.2%, respectively [20]. In Baum’s study, individuals with suspected malaria from the malaria clinic and hospital were included. This may explain why the infection rate determined by Baum et al. was slightly higher than in this current study. However, while highly sensitive, LAMP itself has a limit of detection. Therefore, the number of sub-microscopic infections could be higher than reported in this current finding. If LAMP targets a gene with a higher copy number compared to the 18S ribosomal RNA (*18srRNA*) gene used in this study, this limit of detection may be decreased making a LAMP assay to be more sensitive.

Amongst the low-density asymptomatic cases in this study, the most prevalent species was *P. vivax*. This malaria species distribution is similar to that reported in Imwong’s study conducted in the northwestern border of Thailand [11] and Nguitragool’s study performed in Kanchanaburi and Ratchaburi Provinces in western Thailand [29]. The similarity likely indicates the homogeneity of the distribution of *Plasmodium* species in western Thailand.

Moreover, this current study also observed evidence of heterogeneity of *Plasmodium* prevalence of asymptomatic malaria infections, with some hamlets being completely free of infections, while in other nearby hamlets separated by short distances, approximately 5.57% of the hamlet population was infected. The heterogeneity in the prevalence of malaria infections in this study is in agreement with a previous finding performed in southern Laos PDR [30] and in western Cambodia [31]. Malaria prevalence with spatial heterogeneity between nearby hamlets showed an important variability in *Plasmodium* epidemiology at the community level. The heterogeneity in the *Plasmodium* infection prevalence may at least in part be described by environmental factors, including abundance of mosquito larval habitats, mosquito densities, house location in relation to mosquito breeding grounds, house construction materials and design, and resident protective measures and exposure to mosquitoes due to occupations. It appears that the hamlet with the infected population is a local transmission hotspot that needs special attention by the malaria control programmes.

The protective immunity in malaria-endemic areas correlates with age, which is considered one of the most important factors [32]. Generally, young children are the most susceptible to developing the disease. However, older children and adults are more likely to harbour asymptomatic infections since they might have had several episodes of *Plasmodium* infections and acquired immunity [33, 34]. This current study found that the peak risk for low-density asymptomatic *Plasmodium* infections was in the age group of 31–45 years. A similar peak prevalence in adults has recently been reported in eastern Myanmar which found that all asymptomatic infections were restricted to participants aged > 17 years [35]. This indicates that the infections were more likely transmitted outside of residential areas and younger individuals may have received less vector exposure. Older individuals may have developed enough immune response to suppress the infection allowing it to persist for a period of time without causing any symptoms.

Previously, the study by Zaw et al. conducted in eastern Myanmar found that there was a statistically significant difference in infection rates among occupations. This previous study showed that much higher infection rates were observed in men working in the forests [35]. Consistent with Zaw’s study, a risk factor associated with forest-related activities was observed in this current study since individuals who work on the plantation had significantly increased risk of having asymptomatic sub-microscopic *Plasmodium* infections. Interestingly, a significantly increased risk was also found in merchants, which is consistent with the correlation with extent of outdoor-related activities. Moreover, many past findings have observed that males are more likely to get infected by malaria than females [14, 30, 35]. However, no gender imbalance was observed in this present study. Both males and females are likely to have the same risk of contracting malaria.

In this study, the LAMP malaria assay provides a promising tool for being used in remote field settings, which could render increased efficiency of ongoing malaria control interventions. However, there are some limitations. The false positive could be occurred when using the supernatant, which was not completely clear from the cell pellet especially in blood samples with haemolysis or with high level of lipid in the plasma. The supernatant should be cleared by centrifugation twice before being used. The turbidimetric determination of the LAMP reaction by visual observation is sometimes difficult especially in the weak positive result. The false negative results of LAMP assay could be observed when using the supernatant, which was repeated freezing and thawing or kept on ice for more than 4–5 h. The supernatant should be immediately used to prevent DNA degradation.

## Conclusion

These current findings based on the LAMP survey in a real-world fielding setting reveal substantial sub-microscopic *Plasmodium* carriages among asymptomatic individuals in Tak Province in northwestern Thailand. This study indicates that light microscopy, a rountinely used diagnostic technique, excessively underestimates true malaria parasite prevalence. Thus, it is imperative to employ a higher sensitivity method such as LAMP for parasite field detection in the phase of malaria elimination. These findings also provide an understanding of the current status of asymptomatic sub-microscopic *Plasmodium* infection prevalence, and determine the key demographic characteristics of *Plasmodium* infected cases, such as age groups and occupations. This knowledge could provide crucial information regarding potential infection sources, and could assist in determining the risk of infection within individual hamlets. These findings have important practical implications for targeting activities to eliminate malaria including mass screening and mass anti-malarial drug administration.

## Abbreviations

LAMP: loop-mediated isothermal amplification
RT-LAMP: reverse transcription- LAMP
